# Emerging Alternative Proteinases in APP Metabolism and Alzheimer’s Disease Pathogenesis: A Focus on MT1-MMP and MT5-MMP

**DOI:** 10.3389/fnagi.2019.00244

**Published:** 2019-09-24

**Authors:** Laura García-González, Dominika Pilat, Kévin Baranger, Santiago Rivera

**Affiliations:** Aix-Marseille Univ, CNRS, INP, Inst Neurophysiopathol, Marseille, France

**Keywords:** amyloid precursor protein, matrix metalloproteinases, eta-secretase, meprin-beta, legumain, rhomboid-like protein-4, caspase, neurodegenerative disease

## Abstract

Processing of amyloid beta precursor protein (APP) into amyloid-beta peptide (Aβ) by β-secretase and γ-secretase complex is at the heart of the pathogenesis of Alzheimer’s disease (AD). Targeting this proteolytic pathway effectively reduces/prevents pathology and cognitive decline in preclinical experimental models of the disease, but therapeutic strategies based on secretase activity modifying drugs have so far failed in clinical trials. Although this may raise some doubts on the relevance of β- and γ-secretases as targets, new APP-cleaving enzymes, including meprin-β, legumain (δ-secretase), rhomboid-like protein-4 (RHBDL4), caspases and membrane-type matrix metalloproteinases (MT-MMPs/η-secretases) have confirmed that APP processing remains a solid mechanism in AD pathophysiology. This review will discuss recent findings on the roles of all these proteinases in the nervous system, and in particular on the roles of MT-MMPs, which are at the crossroads of pathological events involving not only amyloidogenesis, but also inflammation and synaptic dysfunctions. Assessing the potential of these emerging proteinases in the Alzheimer’s field opens up new research prospects to improve our knowledge of fundamental mechanisms of the disease and help us establish new therapeutic strategies.

## Alzheimer’s Disease, A Proteolytic Problem

Alzheimer’s disease (AD) is the most common type of neurodegenerative disorder for which only a few drugs have shown transient and moderate anti-symptomatic effects, but there is no treatment that slows down or prevents the progression of the disease. A minority of AD cases find their cause in deterministic genetic mutations in three genes: *PSEN1*, *PSEN2* and *APP*, encoding, respectively, aspartyl proteinase presenilin 1 and 2 (PS1/PS2) and amyloid precursor protein (APP; Van Cauwenberghe et al., 2016). These mutations account for the so-called “familial forms” of the disease. The overwhelming majority of AD cases (∼95%) are sporadic forms of unknown etiology. Despite controversies over the causes of AD, it is still recognized that brain accumulation of the amyloid peptide-β (Aβ) plays a central role in the pathogenic process (Selkoe and Hardy, 2016). Aβ results from the proteolysis of APP, a type I transmembrane protein targeted first at the plasma membrane, then rapidly endocytosed to endosomes to be metabolized to Aβ or subsequently sent to the lysosomal compartment for degradation (Wang X. et al., 2017; Van Acker et al., 2019). Endosomes are thought to be the main locus of Aβ production, which is ensured by canonical β- and γ-secretases (Vassar et al., 1999).

**FIGURE 1:**
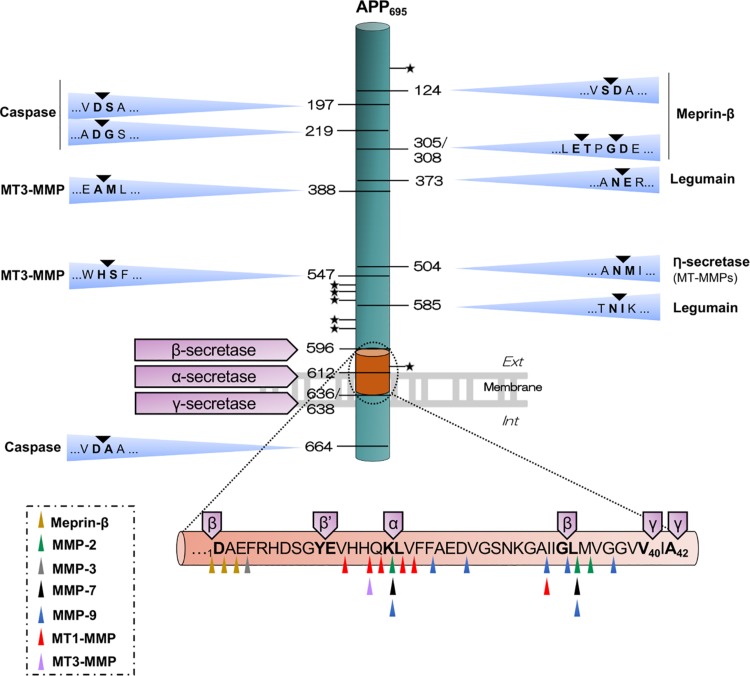
Illustrative outline of the different APP cleavage sites. The scheme shows the canonical (β-, α- and γ-secretases) and non-canonical APP-cleavages that result in the different APP fragments. Meprin-β cleaves APP in five sites, possibly generating soluble APP fragments of different size, sAPPβ or sAPPβ-like (sAPPβ with one or two additional amino acids) and its complementary C-terminal β-CTF or β-CTF-like (β-CTF with one or two amino acids less), which is further processed by γ-secretase, thus producing Aβ_*1–X*_, Aβ_*2–X*_ or Aβ_*3–X*_. Legumain cleaves in two sites, giving rise to two soluble fragments and one C-terminal transmembrane fragment than can be further processed by the canonical enzymes. Caspase can cleave in the N-terminal domain, generating truncated APP-species, and within the intracytoplasmic domain, generating an APP-Ncas fragment and a C-terminal residual peptide of 31 amino acids. RHBDL4 cleavages within the ectodomain at several sites are not yet identified (represented with a star), generating different N- and C-terminal fragments. Cleavage by η-secretase generates a soluble fragment (sAPPη/sAPP95) and a paired transmembrane product η-CTF/CTF-30 that can be further processed by β- or α-secretase to release Aη-β and Aη-α, respectively. MT3-MMP can cleave APP in 4 sites, including the η-secretase site and also within the Aβ sequence. The scheme below represents the Aβ sequence with multiple cleavage sites for various soluble MMPs and MT1-MMP, for meprin-β and for canonical secretases. All the residues are termed using APP_695_ numbering.

β-site APP cleaving enzyme 1 (BACE-1) is the main β-secretase. This type I transmembrane protein of the aspartyl proteinase family needs an acidic environment (optimum pH 4.5) to be enzymatically active (Saric et al., 2013). BACE-1 cleaves numerous substrates, which confers this enzyme a wide spectrum of physiological and pathological activities (Kuhn et al., 2012; Zhou et al., 2012; Vassar et al., 2014), but it is indisputably its ability to process APP that has attracted much attention, especially in relation to AD. As illustrated in Figure 1, BACE-1 cleaves APP between Met_671_ and Asp_672_ to generate the soluble APP-β fragment (sAPPβ) and its complementary C-terminal counterpart of 99 amino acids termed β-CTF or C99. This cleavage signals the first emblematic proteolytic step to the production of Aβ, considered to be one of the main driving forces in AD pathogenesis. BACE-1 can also catalyze amyloidolytic processing by cleavage between Tyr_10_ and Glu_11_ of the Aβ sequence (Huse et al., 2002; Liu et al., 2002; Kimura et al., 2016), the so-called β’-cleavage site (Figure 1). Interestingly, β’-cleavage is favored in the neuroprotective Icelandic APP mutation while the canonic β-cleavage at Asp_1_ is reduced (Kimura et al., 2016). However, a causal effect between β’-cleavage and neuroprotection is not straight forward as Aβ_11–40_ is found in insoluble Aβ pools of post-mortem AD brains (Huse et al., 2002). More recently, it has been shown that BACE-1 can also cleave Aβ_40_ or Aβ_42_ to generate a C-terminal truncated Aβ_34_ form, which appears to be a new biomarker of Aβ clearance in AD as it is noticeably increased in mild cognitive impaired patients along with strong BACE-1 activity (Liebsch et al., 2019). Together, these data confirm early studies (Fluhrer et al., 2003; Shi et al., 2003), placing BACE-1 as a prominent Aβ-generating, but also as an occasional Aβ-“degrading” enzyme under some circumstances. Physiologically relevant Aβ degradation has been mainly attributed to 4 metalloproteinases: neprilysin, insulin degrading enzyme, endothelin converting enzyme and angiotensin converting enzyme, the regulation and functions of which have been extensively reviewed elsewhere (De Strooper, 2010; Nalivaeva et al., 2012). Matrix metalloproteinases (MMPs), including MMP-2, MMP-3, MMP-7 and MMP-9 also cleave within the Aβ sequence (Rivera et al., 2019) and these cleavages have been compared to those of membrane-type MMPs (MT-MMPs) in Figure 1.

The γ-secretase complex is formed by a PS1 or PS2 catalytic subunit and 3 partner proteins, Aph-1, pen-2 and nicastrin (Haass et al., 2012; Rajendran and Annaert, 2012; Masters et al., 2015; Selkoe and Hardy, 2016). Presenilins are acidic proteinases [optimum pH 6.3, (Campbell et al., 2003)] that belong to the family of seven transmembrane domain proteins. Like BACE-1, γ-secretase targets many substrates in addition to APP, with the consequent impact on a vast array of physiological and pathological processes (Haapasalo and Kovacs, 2011). The subcellular location and substrate specificity of γ-secretase may vary depending on the presence of PS1 or PS2 in the complex. While the complex containing PS1 is widely distributed in the cell, a single acidic-dileucine sorting motif present in PS2 directs the γ-secretase complex to late endosomes/lysosomes (Sannerud et al., 2016). γ-secretase performs regulated intramembrane proteolysis to process C99 and release Aβ and the remaining APP intracellular domain (AICD). The latter can be translocated in the nucleus and engages in transcriptional activities, which are to some extent still controversial in the AD field (reviewed in Pardossi-Piquard and Checler, 2012) (Table 1).

**TABLE 1:** Representative of single or combined cleavages of APP by proteinases and known functions of generated fragments.

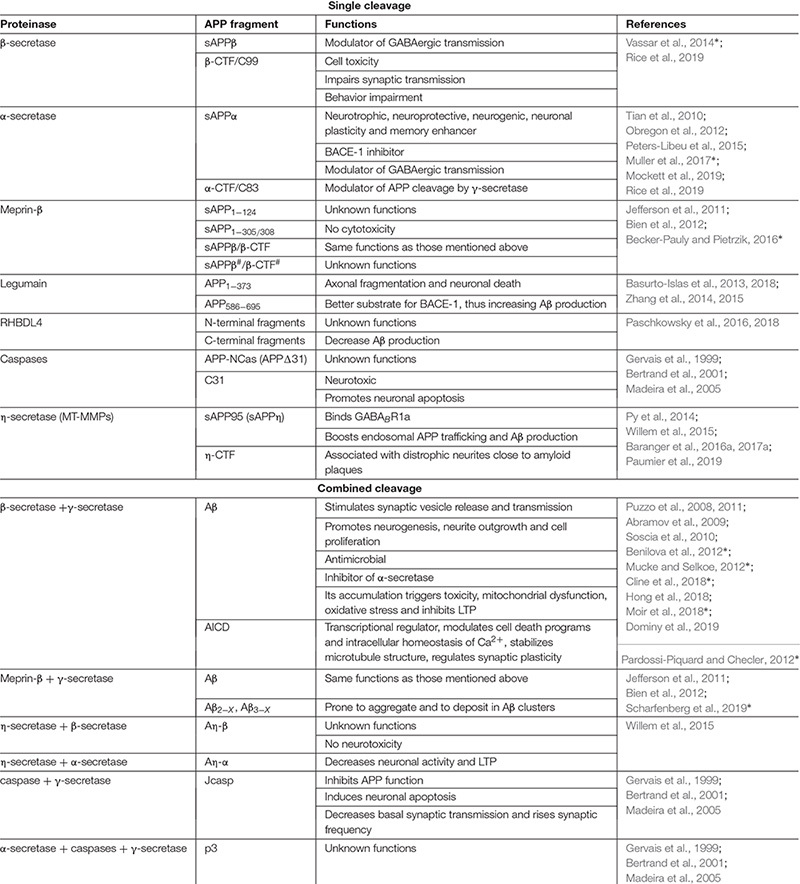

*^∗^The asterisk indicates review articles. #The hash indicates alternative cleavages for meprin-β close to β-secretase site, thus generating a sAPPβ with one or two additional amino acids and its complementary C-terminal β-CTF with one or two less amino acids.*

Along the secretory pathway, APP can also undergo α-secretase cleavage when reaching the plasma membrane. This is performed by a disintegrin and metalloproteinases (ADAMs) of the metzincin superfamily of metalloproteinases (Rivera et al., 2010; Paschkowsky et al., 2019), in a constitutive (ADAM10) or regulated manner (ADAM17; also known as TACE for TNF-α converting enzyme) (Lammich et al., 1999; Jorissen et al., 2010; Kuhn et al., 2010). ADAMs cleave APP in the middle of the Aβ sequence, thereby preventing its generation and releasing at the same time a soluble APP fragment (sAPPα) of ∼110 kDa. In cultured neurons from rodents and humans, sAPPα has shown anti-excitotoxic properties through a mechanism involving the reduction of Ca^2+^ cytotoxic influx (Mattson et al., 1993). These data linking sAPPα to neuroprotection were later confirmed by studies showing that moderate neuronal overexpression of ADAM10 in transgenic AD mice favored an increase in sAPPα levels and a decrease in Aβ, concomitant with a reduction of deficits in long-term potentiation (LTP) and learning (Postina et al., 2004). Likewise, sAPPα could rescue LTP in acute hippocampal slices from mice lacking both APP and its family homolog APP-like protein 2 (APLP2) (Hick et al., 2015), and prevented memory deficits after lentiviral overexpression in the APPswe/PS1dE9 AD mouse model (Tan et al., 2018). While sAPPα release is usually considered as the result of physiological APP processing, Aβ oligomers may be responsible for sAPPα release by cultured neurons and also *in vivo*, which could be considered as a neuroprotective cellular response to the amyloid challenge (Rose et al., 2018). This idea is all the more interesting in the context of data indicating that sAPPα interacts with BACE-1 and inhibits Aβ production (Obregon et al., 2012), in clear contrast with sAPPβ, which fails to interact with BACE-1 because the truncation of 16 aminoacids at the carboxy end (sequence between the α- and β-cleavage sites) is sufficient to impose significant structural changes compared to sAPPα (Peters-Libeu et al., 2015). Recent studies have highlighted new mechanisms by which sAPPα could influence synaptic function through the modulation of the GABA and glutamate neurotransmitter systems. Thus, sAPPα has been shown to act as a ligand of GABA_*B*_R1a -a metabotropic receptor for GABA neurotransmitter-, which results in modulation of hippocampal synaptic plasticity and neurotransmission *in vivo* by decreasing the release of synaptic vesicles (Rice et al., 2019). In addition, sAPPα stimulates trafficking of GluA2-lacking AMPA and NMDA receptors to the synapse, as well as *de novo* protein synthesis of GluA1 protein, thereby providing mechanistic ground for promotion of LTP and synaptic plasticity (Mockett et al., 2019). Consistent with the aforementioned anti-amyloidogenic properties of sAPPα, its complementary C-terminal fragment of 83 amino acids (C83) can also inhibit Aβ generation by interfering with C99 processing by γ-secretase in HEK cells (Tian et al., 2010). The idea that there is cross-regulation between amyloidogenic and non-amyloidogenic pathways is further supported by the fact that Aβ inhibits ADAM10 in mouse primary neuronal cultures, thus favoring endogenous APP processing by β-secretase (Spilman et al., 2016). Since activation and inhibition of ADAMs may be a determining factor for AD pathology, the regulatory potential of the tissue inhibitors of metalloproteinases (TIMPs) should be considered. TIMPs are mainly known as inhibitors of MMPs (see section below), but they also modulate ADAMs activity with low Ki values of 0.5 and 0.1 nM for TIMP-1 and TIMP-3, respectively (Rapti et al., 2008; Baranger et al., 2014). One of our studies shows that TIMP-1 expression is highly upregulated in the brain of transgenic 5xFAD mice at early stages of the pathology and remains durably high, likely matching the progression of neuroinflammation and gliosis (Py et al., 2014). In the diseased brain, reactive astrocytes are the main cellular source of TIMP-1 (Rivera et al., 1997, 2002; Pagenstecher et al., 1998), which in turn promotes astrocyte proliferation (Ogier et al., 2005; Hernandez-Guillamon et al., 2009), suggesting altogether that TIMP-1 could contribute to AD neuroinflammation and/or downregulate ADAM10 activity. As for TIMP-3, one study in neuroblastoma cells reported that this inhibitor not only reduces α-cleavage of APP but also the cell surface levels of ADAM10 and APP, which leads to increased endocytosis and β-secretase cleavage. The potential relevance of TIMP-3 in promoting AD pathogenesis is reinforced in this study by the elevated neuronal immunostaining found in 3xTg transgenic mouse and by increased levels in AD brain homogenates (Hoe et al., 2007). However, another study in 5xFAD mice, showed mild variations of TIMP-3 mRNA levels at different ages, compared to wild type (Py et al., 2014). Clearly, studies are lacking to confirm or refute the idea that a regulatory action of TIMPs on ADAMs could impact on the progression of the disease.

The precise neurotoxicity mechanisms of Aβ are still poorly understood, probably reflecting a combination of events triggered by its accumulation, including stimulation of Tau hyperphosphorylation and subsequent formation of neurofibrillary tangles (NFTs), promotion of inflammation and also synaptic dysfunctions (Benilova et al., 2012; Mucke and Selkoe, 2012; Selkoe and Hardy, 2016; Cline et al., 2018). Monomeric Aβ can be assembled into oligomers, protofibrils or fibrils, the latter being the main constituent of amyloid plaques. Although oligomers are increasingly considered to be the most toxic Aβ assemblage, recent data suggest that only a limited fraction of highly diffusible small oligomers have adverse effects, for instance on the disruption of synaptic activity (Hong et al., 2018). Missing relevant Aβ structures as possible therapeutic targets might be one of the reasons why anti-Aβ strategies developed to fight AD have not yet demonstrated significant clinical benefits (Panza et al., 2019). In the case of therapies based on β- and γ-secretase inhibitors, a possible cause of clinical failure could be the difficulty of inhibiting these proteinases without causing side effects resulting from non-targeted proteolytic inhibition on their many physiological substrates (Karran et al., 2011; Vassar et al., 2014; De Strooper and Chavez Gutierrez, 2015; Barao et al., 2016; Ohno, 2016; Yan, 2017). Besides Aβ, other potential neurotoxic APP metabolites generated with the contribution of BACE-1 activity were initially reported. This was the case of a 35 kDa sAPP NTF fragment (1-286) and sAPPβ whose binding to death receptor 6 (DR6) induced axonal pruning and neuronal apoptosis mediated by caspase activation (Nikolaev et al., 2009). The implication of this 35 kDa fragment was later refuted; instead sAPPβ was reported to induce axonal pruning but not neuronal death, and to interact with DR6 through the more C-terminal E2 domain placed above the β-cleavage site (Olsen et al., 2014). Furthermore, the involvement of DR6 in Alzheimer’s neurodegeneration could not be proved in two transgenic mouse models of AD deficient for DR6 (Kallop et al., 2014). The roles of C-terminal APP fragments resulting from the proteolytic activity of canonical secretases have also been investigated. Thus, many studies using genetic or pharmacological approaches to increase C99 levels in animal models consistently demonstrate the pathogenic properties of C99 linked with AD phenotypes (Neve et al., 1996; Oster-Granite et al., 1996; Song et al., 1998; Matsumoto et al., 2002; Lauritzen et al., 2012, 2016; Bourgeois et al., 2018). It is noteworthy that C99 accumulates in the brains of AD patients (Pera et al., 2013; Kim et al., 2016) and shows a better correlation than Aβ with the degree of vulnerability to neurodegeneration (Pulina et al., 2019). The other C-terminal APP fragment that has focused a great deal of research activity in recent years is the AICD. Despite controversial data has been often reported on its physiological and pathological functions, AICD has gained consensus as signaling molecule that can translocate to the nucleus and regulate the expression of manifold genes, some of them involved in AD pathogenic mechanisms (Kim et al., 2003; Chang et al., 2006), but also in cytoskeleton dynamics, cell cycle control or synaptic plasticity. A recent review has identified nearly 40 genes targeted by the AICD in relation with these and other processes (Bukhari et al., 2017). These genes will need to be tested for their possible contribution to signaling pathways that depend on APP and their consequent impact on brain pathology and physiology.

Other APP fragments generated by emerging APP processing enzymes are currently being investigated to determine their potential physiological or pathological actions and they will be discussed below.

## Insufficiently Explored Territories

The idea that APP metabolites other than Aβ could actually impact on AD pathology is gaining momentum. However, the assessment of their physiological functions should also be carried out in parallel in order to have a more accurate picture of the pathophysiological scenario. The same holds true for Aβ, which remains the main driving force behind AD pathology, but whose physiological activities are still poorly understood and certainly insufficiently studied. Thus, for example, a few studies indicate that Aβ has antimicrobial activities, leading some authors to propose that it could act as an innate immunity protein in the brain (Soscia et al., 2010; Moir et al., 2018; Dominy et al., 2019). In addition, Aβ has been shown to be a positive modulator of synaptic vesicle release (Abramov et al., 2009) and synaptic transmission (Puzzo et al., 2008, 2011). In fact, picomolar concentrations of Aβ are sufficient to stimulate nicotinic receptors and subsequently LTP and learning and memory in healthy rodents (Puzzo et al., 2008, 2012; Morley et al., 2010). To what extent the failure of current anti-Aβ therapies could be due to interference with these (or other unknown) physiological roles of Aβ, remains an open question.

Despite the importance of APP proteolysis and the resulting fragments in the context of AD, APP role as a cell adhesion protein or receptor also deserves consideration insofar as the alteration of these functions may indeed contribute to the disease process. Accordingly, it is proposed that APP contributes to cell adhesion responsible for maintaining the structure of synapses and neural circuits through its interactions with extracellular matrix and adhesion molecules, but also through its own dimerization at the cell surface (see van der Kant and Goldstein, 2015; Montagna et al., 2017; Muller et al., 2017; Sosa et al., 2017), which is regulated, *inter alia*, by APP binding with Cu^2+^ and Zn^2+^ (Wild et al., 2017). Disruptions of physiological functions of APP could cause synaptic dysfunctions that occur in the early phases of AD, but it could also alter the subcellular location of APP and thus the degree of pathogenicity of proteolytic fragments depending on whether they are mainly generated in endosomes (e.g., C99, Aβ) or in the cell membrane (e.g., sAPPα). APP has also been described as a functional cellular receptor for Aβ to the point that Aβ oligomers isolated from AD brains require APP expression to induce synaptotoxic effects on mice hippocampi (Wang Z. et al., 2017). By the same time, it was shown that Aβ oligomers and also Tau could cause APP-dependent impairment of LTP and spatial learning in mice (Puzzo et al., 2017). Another work highlights the potential of full length APP as functional receptor for the regulation of cholesterol/lipoprotein metabolism and Aβ clearance (Fong et al., 2018). In this case, human astrocytes derived from induced pluripotent stem cells (iPS) deficient for APP exhibited reduced levels of intracellular cholesterol, as well as a reduced ability to endocyte apolipoprotein E (APOE) and Aβ, which are major processes disturbed in AD and possible cause of deficient Aβ clearance. Similar deficits have been observed in astrocytes expressing *APP* harboring the Swedish familial mutation, characterized by reduced canonical levels of APP due to exacerbated cleavage of APP by β-secretase. It is interesting to note that the inhibition of β-secretase reversed these effects, thereby highlighting the potential benefits of such inhibition specifically in astrocytes (Fong et al., 2018). These few examples illustrate the importance of canonical APP as a regulator of tissue homeostasis and the extent to which subtle alterations in its biology could trigger or accompany pathological processes. Further research will be needed to increase our knowledge of the biology of APP and thus determine whether maintaining APP levels could be a therapeutic strategy of interest.

The complexity of interactions between APP and canonical secretases results in multifunctional derivatives of APP (Chen et al., 2015; Liu et al., 2019). The emergence of proteinases with new APP transformation activities and new fragments does not precisely simplify this scenario, but may be useful to broaden the spectrum of mechanisms to be targeted in a therapeutic context. We will focus our attention in the following section on a subfamily of MMPs, the MT-MMPs, but will also discuss recent findings linking meprin-β, legumain, rhomboid-like protein-4 (RHBDL4) and caspases to APP/Aβ metabolism (Figure 1 and Table 1).

## Some Emerging APP Processing Proteinases

### Meprin-β

Meprin-β is a type I transmembrane protein of the astacin group of the metzincin superfamily of metalloproteinases (Villa et al., 2003). The enzyme is naturally inhibited by fetuin-A (or alpha2-Heremans-Schmid glycoprotein) and fetuin-B, but not by cystatin C, a closely related inhibitor (Hedrich et al., 2010; Karmilin et al., 2019). Meprin-β activity has been linked to the processing of a variety of substrates, including inflammatory cytokines, and cell adhesion and extracellular matrix molecules in different organs. Overall, this proteinase exerts control on inflammatory/immune and cell migration processes. In the nervous system, meprin-β has been described as a new alternative APP processing enzyme, which links its activity to AD (reviewed in Scharfenberg et al., 2019). In this context, several studies have reported increased mRNA and protein levels of meprin-β in AD patients compared to age-matched healthy individuals (Bien et al., 2012; Schlenzig et al., 2018). Produced by brain neurons, meprin-β exists in plasma membrane-bound form or in soluble form after shedding by ADAM10 or ADAM17. Using a synthetic peptide mimicking APP/Aβ sequence, it was shown *in vitro* that meprin-β was able to cleave APP at the β-cleavage site and after the first and second amino acids of the Aβ sequence, suggesting that meprin-β can behave as a β-secretase. This was further supported by the fact that meprin-β expression in BACE-1/2 knockout fibroblasts was sufficient to generate Aβ (Bien et al., 2012). In addition, meprin-β can cleave APP near the N-terminal end to generate truncated fragments of 11 and 20 kDa (N-APP), as demonstrated by terminal amine isotopic labeling of substrates. N-APP were detected in mice brains as well as in the brains of healthy individuals and patients with AD, but not in meprin-β knockout mice, validating the role of meprin-β in the physiological processing of APP (Jefferson et al., 2011). These APP fragments did not induce toxicity in neuronal cultures and therefore further work will be needed to elucidate their physiological functions. Unlike BACE-1, meprin-β cleaves APP on the plasma membrane before it undergoes endocytosis, and preferentially generates Aβ_1–40_ and a N-terminally truncated Aβ (Aβ_2–X_), which is more prone to aggregation than Aβ_1–40_. This is of interest, as truncated Aβ species may promote the formation of highly toxic Aβ oligomers. Also important, Aβ_2–X_ can only be generated by membrane-bound meprin-β and not by the soluble form after ADAM10/17-mediated shedding (Bien et al., 2012). An additional regulatory interplay between meprin-β and α-secretase has been identified; meprin-β deletion in mice correlates with higher levels of sAPPα, thereby suggesting *in vivo* competition with α-secretase for membrane-bound APP (Schonherr et al., 2016). It has to be noted that *APP* mutations proximal to the β-cleavage site, like the Swedish (K_670_N-M_671_L) or the “protective” Icelandic (A_673_T), prevent the generation of Aβ_2–X_ by meprin-β as opposed to the lack of effect of distal mutations (i.e., London (V_717_I), highlighting the importance for meprin-β of aminoacid composition around the β-cleavage site (Schonherr et al., 2016). Overall, meprin-β bears great interest as alternative APP processing enzyme with amyloidogenic features. It remains to be determined to which extent the relatively small amount of Aβ_2–X_ generated in AD compared to Aβ42 provides meprin-β with a relevant pathogenic role in AD. Further investigations will also be necessary to assess the possible impact of meprin-β as a regulatory factor of neuroinflammatory processes operating in AD and other brain disorders.

### Legumain

Legumain, also known as asparagine endopeptidase, is a soluble cysteine proteinase mainly found in endo-lysosomal compartments that provide the optimal functional pH of 6. Its proteolytic activity is inhibited by cystatin C and closely related cystatins E/M and F (Chen et al., 1997; Alvarez-Fernandez et al., 1999). The enzyme was later renamed δ-secretase (Zhang et al., 2015) to highlight its role in APP cleavage at Asn_586_ that generates the δ-CTF first identified in the nineties (Simons et al., 1996; Scharfenberg et al., 2019). As lysosomal dysfunction is a transversal pathogenic mechanism, legumain has been involved in numerous pathological settings, including atherosclerosis, osteoporosis, cancer, ischemic stroke, and neurodegenerative diseases (Lunde et al., 2019). Legumain has been described in relation with AD as a modulator of Tau phosphorylation (Basurto-Islas et al., 2013), and as a Tau- or APP-cleaving enzyme (Zhang et al., 2014, 2015). Under acidic conditions (e.g., brain ischemia, hypoxia, or AD), legumain can translocate from neuronal lysosomes into the cytoplasm, where it cleaves I_2_^*PP2A*^ (also known as SET). This cleavage generates two fragments, I_2_^*NTF*^ and I_2_^*CTF*^ that inhibit protein phosphatase 2A (PP2A), a key phosphatase that limits Tau hyperphosphorylation both *in vitro* (Basurto-Islas et al., 2013) and *in vivo* (Basurto-Islas et al., 2018). In total, legumain activity promotes the hyperphosphorylation of Tau protein, which is a major hallmark of AD pathogenesis.

In addition to controlling Tau phosphorylation, legumain generates neurotoxic fragments after cleavage of Tau and APP. The brain of AD patients show increased levels of the Tau_1–368_ fragment compared to healthy individuals, in correlation with increased levels of legumain activity (Zhang et al., 2014). This study showed that Tau_1–368_ generated by legumain inhibits microtubule polymerization *in vitro* and promotes apoptosis in rat cultured neurons. Consistent with these *in vitro* findings, the deletion of legumain in the Tau P301S transgenic mouse model of AD prevents synaptic dysfunction and improves learning and memory. Furthermore, mice virally infected with uncleavable Tau mutant showed reduced pathological and behavioral defects as compared with mice infected with Tau P301S. Of note, antibodies specifically raised against the Tau_1–368_ neoepitope were found in AD brains and absent in legumain knockout mice, thus validating *in vivo* legumain-mediated Tau processing (Zhang et al., 2014). The same group also demonstrated that legumain can cleave APP at positions 373 and 585 (APP_695_ numbering), generating 2 fragments with distinctive cytotoxicity (Zhang et al., 2015). While APP_1–373_ triggers axonal fragmentation and neuronal death in primary cultured neurons, the APP_586–695_ fragment appears to be a better pro-amyloidogenic substrate for BACE-1 than full-length APP and the other legumain-derived C-terminal fragment (APP_374–695_). In support of these *in vitro* data, legumain deficiency in the 5xFAD transgenic mouse model of AD (Oakley et al., 2006) causes a drop of Aβ_40_, Aβ_42_ and amyloid plaque burden, while it increases spine density, and prevents deficits in LTP and learning and memory (Zhang et al., 2015). The first step for pharmacological validation of legumain in AD was obtained using a chemical inhibitor, which reduced the formation of neurotoxic Tau fragments and Aβ accumulation in P301S and 5xFAD mouse models, respectively. In addition, legumain inhibition reduced synaptic loss, improved LTP, prevented microglial activation and reduced the levels of inflammatory mediators TNF-α and IL-1β (Zhang et al., 2017).

### Rhomboid-Like Protein-4

There are five active intramembrane proteinases Rhomboids in humans, RHBDL1-4 and PARL. RHBDL1-4 are located in the secretory pathway, while PARL is found in mitochondria. This family of serine proteinases is involved in many cellular processes, such as inflammatory signaling, cell migration, proliferation and mitochondria homeostasis (reviewed in Dusterhoft et al., 2017; Paschkowsky et al., 2019). Recently, Paschkowsky et al. (2016) showed in HEK cells expressing APP_695_ that RHBDL4 but not RHDBL1, 2 and 3, cleaves at multiple sites within the ectodomain of APP (Figure 1) and APLP1 and APLP2, generating a N-terminal fragment of ∼70 kDa and different C-terminal fragments whose functions are still unknown. The use of specific inhibitors for a wide spectrum of proteinases, including BACE-1, α- and γ-secretase, aspartyl and cysteine proteinases, and metalloproteinases did not modify the APP fragmentation profile generated by RHBDL4, suggesting a specific action of this proteinase. Moreover, it was shown that RHBDL4 activity decreases the levels of Aβ_38_, Aβ_40_ and Aβ_42_. RHBDL4 is mainly located in the endoplasmic reticulum, which contains low levels of membrane cholesterol. Interestingly, RHBDL4-mediated cleavage of APP is negatively regulated by cholesterol when it binds to two transmembrane domains of the proteinase, but not to APP. Thus, it is possible that lowering cholesterol could increase RHBDL4 activity on full-length APP, with the consequent generation of CTFs and the decrease in Aβ generation (Paschkowsky et al., 2018). Overall, these data underline the unexpected important role of RHBDL4, which is not a classical secretase, in the metabolism of APP/Aβ. Nevertheless, much remains to be done to validate most of the referred *in vitro* observations in *in vivo* models with relevance for AD pathogenesis.

### Caspases

Caspases are pleiotropic cysteine proteinases that specifically cleave target proteins after an aspartic acid residue (Hyman and Yuan, 2012). Several studies have demonstrated in a variety of culture cell systems the ability of caspase-3, -6 and -8 to cleave APP, mainly between amino acids V_664_-D_665_ (APP_695_ numbering) within the intracytoplasmic domain (Gervais et al., 1999; LeBlanc et al., 1999; Pellegrini et al., 1999; Weidemann et al., 1999). Cleavage of APP by caspases generates a membrane bound N-terminal fragment, named APP-Ncas, and an intracellular C-terminal peptide of 31 amino acids, named APP-Ccas or C31. When γ-secretase processes C99 into Aβ, the remaining AICD is further cleaved by caspase to generate C31 and JCasp fragments, both of which promote neuronal apoptosis (Bertrand et al., 2001; Madeira et al., 2005). For this reason, it was suggested that the cytotoxicity of C99 might actually result from subsequent formation of C31 by caspases (Lu et al., 2000). It is also noteworthy that AD brains show elevated levels of caspases and that APP fragments resulting from caspase cleavage colocalize with amyloid plaques (Gervais et al., 1999; Lu et al., 2000). Eventually, caspase-mediated APP cleavage stimulates Aβ production in B103 and NT2 cells (Gervais et al., 1999). However, other authors have challenged these data in a study where caspase removed the internalization signal (YENPTY) located in the extremity of the AICD, thereby leading to impaired APP internalization and Aβ production in B103 cells (Soriano et al., 2001). These results were recently confirmed using CRISPR/Cas9 editing in human iPS to remove the last 36 amino acids of APP containing the internalization motif. The expression of this C-terminal truncated APP in human iPS-derived neurons prevented the production sAPPβ and Aβ, while stimulating α-secretase cleavage. In addition, it was shown that cultured hippocampal neurons with truncated *APP* gene accumulate APP on the plasma membrane and present reduced colocalization of BACE-1 and APP in endosomes, confirming a key role of the YENPTY sequence in APP internalization and Aβ production by endosomes (Sun et al., 2019).

Caspases have also been proposed as a possible molecular link between amyloid and Tau pathologies in AD, since Aβ simulation of neuronal apoptosis is accompanied by an increase of Tau cleavage by caspases at Asp_421_. This is consistent with the observation that C-terminal truncated forms of Tau show increased polymerization capacity *in vitro* and are associated with NFTs formation in the brain of AD patients (Gamblin et al., 2003; Rissman et al., 2004).

### MT-MMPs

MT-MMPs are new players in the field of AD, mainly because of their ability to regulate APP metabolism and therefore amyloidogenesis. In the next sections, we will focus on two MT-MMPs, MT1-MMP and MT5-MMP, which have been associated with the pathophysiological mechanisms that support AD. More generally, the involvement of other MMPs in AD and other neurodegenerative disorders has been discussed elsewhere (Rivera et al., 2019).

#### Structure of MT1-MMP and MT5-MMP

MMPs constitute a multigenic family of 24 endopeptidases that belong to the metzincin superfamily of metalloproteinases. MMPs show pleiotropic regulatory actions in many processes in the nervous system, including axonal growth (Pastrana et al., 2006; Ould-yahoui et al., 2009; Trivedi et al., 2019), neurogenesis (Wojcik et al., 2009), learning and memory (Beroun et al., 2019), glial reactivity and inflammation (Chopra et al., 2019; Montaner et al., 2019; Muri et al., 2019), cell migration (Ould-Yahoui et al., 2013) or neuronal death (Gu et al., 2002; Jourquin et al., 2003). The many aspects of MMP functions and action mechanisms in the nervous system have been extensively discussed earlier (Rivera et al., 2010, 2019; Baranger et al., 2014).

MMPs are mostly secreted proteinases, but 6 transmembrane proteins form the so-called subfamily of MT-MMPs: MT1-MMP (MMP-14), MT2-MMP (MMP-15), MT3-MMP (MMP-16), MT4-MMP (MMP-17), MT5-MMP (MMP-24) and MT6-MMP (MMP-25). All MT-MMPs share closely related structural features, described in detail elsewhere (Itoh, 2015). These include a signal peptide that targets the MT-MMP to the endoplasmic reticulum, where it is proteolytically excised, and then a pro-domain bearing a conserved cysteine that interacts with the catalytic domain and maintains the enzyme as an inactive zymogen. The pro-peptide can be separated from the rest of the molecule by redox-mediated chemical reactions or after proteolytic cleavage by serine endoproteinase furin. This mechanism, called “cysteine switch,” implies the dissociation between the Cys residue of the pro-peptide and the Zn^2+^ of the catalytic domain, which leads to enzymatic activation. The catalytic domain is highly conserved domain across MT-MMPs (*de facto*, across MMPs) and is linked by a hinge region to the hemopexin domain, which has a more variable sequence, thus conferring certain specificity to the binding of substrates and endogenous TIMPs. MT-MMPs are linked to the plasma membrane either by a glycosylphosphatidyl inositol (GPI) bond (MT2-MMP and MT6-MMP) or by a transmembrane domain (MT1-, MT2-, MT3- and MT5-MMP), followed in this case by an intracytoplasmic domain that can control MT-MMP cell traffic and its proteolytic activity (Uekita et al., 2001; Wang et al., 2004; Sakamoto and Seiki, 2009). Analysis of the amino acid sequence reveals a variable percentage of sequence identity between human MT1- and MT5-MMP, ordered from N- to C-terminus as follows: pro-domain (44%), catalytic (72%), hinge (41%), hemopexin (66%), stem part (17%), transmembrane (37%) and intracytoplasmic (20%). Despite the high degree of sequence identity between MT1- and MT5-MMP, human and mouse MT5-MMP carry unique dibasic motifs in their stem regions, which are recognized by proteinases from the furin proprotein convertase family. Proteolytic cleavage by furin gives MT5-MMP singular biochemical properties, in particular an ephemeral presence on the plasma membrane since it is released into the medium in soluble form (Pei, 1999; Wang and Pei, 2001).

#### Physiological Functions of MT1- and MT5-MMP

##### MT1-MMP

MT1-MMP is ubiquitously distributed in the body. In the nervous system it is mainly expressed by microglia, astrocytes and neurons (Liao and Van Nostrand, 2010; Langenfurth et al., 2014; Py et al., 2014; Itoh, 2015). As most MMPs, MT1-MMP degrade extracellular matrix (ECM) proteins, in particular type I and III collagen, fibronectin, laminin, vitronectin and proteoglycans (Ohuchi et al., 1997). MT1-MMP catalyzes the conversion of pro-MMP-2 into the active MMP-2 form through a mechanism involving one of its endogenous inhibitors (see below) (Sato et al., 1996; Llano et al., 1999; Pei, 1999; Baranger et al., 2014). MT1-MMP is the most studied of the MT-MMPs and the progressive identification of new substrates beyond ECM has led to an expansion of the biological functions of the proteinase. MT1-MMP has an impact on inflammatory processes by modulating the action of cytokines, chemokines, proteinase inhibitors or receptors. For example, MT1-MMP can activate pro-TNF-α into active TNF-α, and inactivate CXCL12 (SDF1α), as well as complement protein C3, secretory leukocyte proteinase inhibitor (SLPI), IL-8 and other chemokines. Cellular receptors and membrane proteins such as DR6, the VEGF receptor neuropilin, the prion protein or a ligand of Notch receptor Delta-like I, are also processed by MT1-MMP (Overall et al., 2004; Tam et al., 2004; Jin et al., 2011; Starr et al., 2012; Kojima et al., 2014; Itoh, 2015). MT1-MMP knockout mice are not viable beyond one month of age, suggesting an important role of this proteinase in development (Holmbeck et al., 1999). In this context, it is now well documented that MT1-MMP contributes to cell migration including monocytes/macrophages, endothelial cells or neural stem cells (Galvez et al., 2001; Matias-Roman et al., 2005; Ould-Yahoui et al., 2013). It is noteworthy that in some cases the stimulation of migration or chemotaxis by MT1-MMP does not depend on its catalytic activity, but rather on the properties of its cytoplasmic tail. Thus, proteolytic inhibition of MT1-MMP does not affect migration of isolated macrophages on matrigel invasion tests. In addition, the expression of catalytically active and inactive forms of MT1-MMP in MT1-MMP knockout macrophages rescue chemotaxis properties, while a mutant form lacking the cytoplasmic tail does not (Sakamoto and Seiki, 2009). Recently, Aguirre and collaborators elegantly demonstrated that MT1-MMP is crucial in the inflammatory response upon intraperitoneal LPS injection in wild type and MT1-MMP knockout pups (5–8 days post-natal). Under these experimental conditions, they observed that the absence of MT1-MMP reduces life span, which is associated with lung enlargement and higher neutrophil recruitment (Aguirre et al., 2017). The effects of MT1-MMP appear to be related to MMP-2 activation, as lower concentrations of active MMP-2 are found in MT1-MMP knockout pups, associated with higher levels of MMP-2 substrate S100A9 (Aguirre et al., 2017). The latter is an inflammatory protein (Vogl et al., 2012) also implicated in AD pathogenesis (Chang et al., 2012). Taken together, these results clearly reflect the multifaceted mode of action of MT1-MMP, with functions that depend on proteolysis, but also on protein-protein interactions mediated by the C-terminal intracytoplasmic tail. It remains to be demonstrated whether this functional diversity is limited to certain cell types or organs, in particular with regard to the role of MT1-MMP in the nervous system.

##### MT5-MMP

To date, fewer substrates have been described for MT5-MMP, compared to MT1-MMP (reviewed in Itoh, 2015). MT5-MMP preferentially cleaves proteoglycans and to a lesser extent fibronectin, but not type I collagen or laminin (Wang et al., 1999). MT5-MMP expression is mainly confined to the nervous system, with levels in rodents reaching their maximum before birth and remaining high into adulthood in areas like the hippocampus or the cerebellum (Pei, 1999; Jaworski, 2000; Hayashita-Kinoh et al., 2001; Sekine-Aizawa et al., 2001; Warren et al., 2012). Such a distribution is consistent with the proposed role of MT5-MMP in brain development and more broadly in neural plasticity. Similarly, MT5-MMP has been reported to promote axonal growth (Hayashita-Kinoh et al., 2001) and to control the activation of adult neural stem cells under physiological and regenerative conditions by a mechanism that requires the cleavage of N-cadherin (Porlan et al., 2014), one of the non-ECM substrates of MT5-MMP. Despite the presumed developmental role of MT5-MMP, knockout mice are viable and have no apparent phenotypes, indicating that there is functional redundancy with other proteinases in physiological conditions. However, phenotypes linked to MT5-MMP deficiency become evident when mice are subjected to stressful conditions. This is for instance the case after sciatic nerve injury, where MT5-MMP deletion prevents aberrant sprouting of nociceptive Aβ-fibers and the resulting mechanical allodynia (Komori et al., 2004). The implication of MT5-MMP in post-lesion axonal regeneration has also been suggested in the context of concerted work with ADAM10 in reactive astrocytes to control post-lesion synaptic remodeling (Warren et al., 2012). We will see later that the combined action of MT5-MMP and ADAM10 has also been proposed in AD, with different functional implications. Deletion of MT5-MMP also revealed a reduction in hyperalgesia after intraplantar injections of IL-1β or TNF-α in a model of thermal pain, indicating that MT5-MMP plays role in the inflammatory pathways driven by these cytokines. The proposed mechanism associates the absence of MT5-MMP with deficient cleavage of its substrate N-cadherin, a cell adhesion molecule involved in synapse architecture. N-cadherin cleavage is required to ensure communication between mast cells and sensory neurons for the transmission of nociceptive stimuli (Folgueras et al., 2009). It is therefore concluded that MT5-MMP is capable of regulating the neuroinflammatory process (Baranger et al., 2016a).

The implication of MT5-MMP in synaptic processes has been suggested by the discovery that the proteinase interacts with the AMPA binding protein and the glutamate receptor-interacting protein. Both are postsynaptic density-95/Discs large/zona occludens-1 (PDZ) protein containing domains. The interactions between these PDZ proteins and MT5-MMP are mediated by the last three carboxyl terminus amino acids of the proteinase, thus facilitating its targeting to synapses and the cleavage of N-cadherin (Monea et al., 2006). In addition, it has been reported that MT5-MMP could act in synergy with γ-secretase to affect synaptic protein levels of GluA2 and PSD95, eventually resulting in a decrease in synaptic transmission (Restituito et al., 2011); however, functional evidence of such interaction is still lacking.

MT5-MMP processing and metabolism are tightly controlled; before reaching the plasma membrane, MT5-MMP can be cleaved by a furin-like activity in its stem part, leading to generation of a catalytically active soluble form of the enzyme that lacks the C-terminal part (Wang and Pei, 2001). Once on the membrane, MT5-MMP can be internalized in the endosomes, where it interacts with the PDZ Mint3 protein, allowing proteinase to be recycled into the membrane (Wang et al., 2004). Despite having the structure of a membrane protein, MT5-MMP can mostly be found in intracellular and extracellular compartments due to its shedding and cellular routing.

##### TIMPs

As with the other members of the MMP family, TIMPs control the catalytic activities of MT1- and MT5-MMP. Of the four TIMPs, TIMP-2, -3 and -4 inhibit MT1-MMP, while MT5-MMP is only targeted by TIMP-2. Both MT-MMPs can activate MMP-2 through a process that requires the formation of a tripartite molecular complex formed by two MT-MMPs, a TIMP-2 and a pro-MMP-2; the N-terminal domain of TIMP-2 interacts with MT1-MMP on the plasma membrane, while its C-terminal domain binds to the hemopexin domain of pro-MMP-2, then the pro-peptide of pro-MMP-2 is readily removed by an adjacent MT1-MMP eventually resulting in MMP-2 activation (Strongin et al., 1995). It should be noted that TIMP-1, which targets soluble MMPs and ADAM10, is a very poor inhibitor of MT-MMPs (reviewed in Baranger et al., 2014).

#### MT1-MMP and MT5-MMP Contribute to Alzheimer’s Pathogenesis

##### MT1-MMP

Higashi and Miyazaki were the first to show that MT1-MMP could cleave APP between residues Asn_579_ and Met_580_ (VLAN_579_-M_580_ISEPR) of APP_770_ after being activated by concanavalin A in the HT1080 fibrosarcoma cell line (Higashi and Miyazaki, 2003b). Three years later, Ahmad and collaborators showed that MT1-, MT3-, and MT5-MMP, but not MT2-, MT4- and MT6-MMP were able to cleave APP_770_ when co-expressed in HEK293 cells (Ahmad et al., 2006). Mass spectrometry analysis for MT3-MMP cleavage-sites revealed the same VLAN_579_-M_580_ISEPR site previously identified for MT1-MMP and 3 other cleavage sites at Ala_463_-Met_464_, His_622_-Ser_623_ and His_685_-Gln_686_ (Higashi and Miyazaki, 2003b), the latter being located in the Aβ sequence, just upstream of the α-cleavage site (Figure 1). Despite this, Aβ levels remained stable in HEK293 cells expressing MT3-MMP, probably indicating that MT3-MMP processes APP in cell compartments that do not influence amyloidogenesis. Although the APP cleavage sites for MT1-MMP and MT5-MMP were not specifically identified in the Ahmad study, the analysis of the APP degradation profile by western blot suggested that both MT-MMPs should cleave APP at the same location as MT3-MMP (Ahmad et al., 2006). Unlike MT3-MMP, the functional interaction between MT1-MMP and Aβ was shown when MT1-MMP overexpressed in COS cells degraded exogenous Aβ. In addition, the recombinant catalytic domain of MT1-MMP could degrade amyloid plaques when incubated *ex vivo* on brain slices of transgenic AD Tg2576 mice (Liao and Van Nostrand, 2010). In this way, MT1-MMP joins the list of metalloproteinases with Aβ-degrading, activity, such as MMP-2, MMP-3, MMP-7 and MMP-9 (Roher et al., 1994; Backstrom et al., 1996; Deb et al., 2003; White et al., 2006; Yan et al., 2006; Yin et al., 2006; Hernandez-Guillamon et al., 2010, 2015; Liao and Van Nostrand, 2010; Py et al., 2014; Brkic et al., 2015; Taniguchi et al., 2017; Rivera et al., 2019) (Figure 1). In complement of these results, we have recently reported that MT1-MMP can also prominently increase Aβ levels in the HEK293 cell model of amyloidogenesis, which stably expresses *APP* with the familial Swedish mutation (HEKswe) (Paumier et al., 2019). Transiently expressed MT1-MMP in HEKswe interacts with APP and induces the release of a soluble APP fragment of ∼95 kDa (sAPP95), distinct of sAPPα or sAPPβ generated by α- and β-secretase, respectively. The complementary transmembrane fragment of ∼30 kDa (CTF-30) and C99 are also dramatically increased and their levels highly correlated, suggesting that CTF-30 may be the precursor of C99 (Paumier et al., 2019). It is also noteworthy that MT1-MMP content is strongly upregulated in the cortex and hippocampus of 6-month old 5xFAD mice (Py et al., 2014). This mouse model mimics the symptoms and pathology of AD, including exacerbated amyloidosis, neuroinflammation and synaptotoxicity (Oakley et al., 2006), deficits in LTP (Crouzin et al., 2013) and cognition (Girard et al., 2013, 2014; Giannoni et al., 2016; Baranger et al., 2017a, b), and eventually neuronal death (Oakley et al., 2006; Jawhar et al., 2012; Eimer and Vassar, 2013). MT1-MMP upregulation in 5xFAD mice timely and spatially correlates with the increase in C99 levels (Py et al., 2014), thus providing indirect *in vivo* support for the pro-amyloidogenic features of this MMP described in HEKswe cells (Paumier et al., 2019).

Although functional interactions between MT1-MMP and APP/Aβ are not fully understood, it has been shown that the release of sAPP95 by MT1-MMP does not involve BACE-1, but it may involve MMP-2. It is estimated that about 50% of the sAPP95 released after MT1-MMP expression in HEKswe cells results from concerted action with MMP-2, MT1-MMP being in any case the limiting factor in this enzymatic tandem (Paumier et al., 2019). A plausible scenario would be that activation of MMP-2 by MT1-MMP (Sato et al., 1996) allows MMP-2 access to membrane substrates (i.e., APP), which would not otherwise be accessible for this soluble MMP. MMP-2, mainly considered as a soluble Aβ-degrading proteinase (Yin et al., 2006), could thus extend its range of action to the pericellular environment thanks to membrane docking provided by MT1-MMP. Another interesting feature linking MT1-MMP, MMP-2 and APP is that MT1-MMP processing of APP releases a soluble APP fragment (likely sAPP95) lacking a potent inhibitor of MMP-2 activity (Miyazaki et al., 1993). In this study, purified soluble forms of APP could efficiently inhibit MMP-2 degradation of gelatin as well as Aβ cleavage between K_16_-L_17_ (α-cleaving site) (Miyazaki et al., 1993). Years later, the authors identified a decapeptide sequence ISYGNDALMP, called APP-derived peptide inhibitor (APP-IP), located 6 amino acids downstream the cleavage site (N_504_-M_505_ISEPRISYGNDALMP) shared by MT1-MMP and MT3-MMP (and also MT5-MMP, see below). APP-IP shows low nanomolar range potency toward MMP-2 (IC_50_ value of 30 nM) and is a weaker inhibitor for MT1-MMP, MMP-3, -7 and -9, with IC_50_ values between 2 and 10 μM. Although sAPPα contains the decapeptide, it appears to be a weaker MMP-2 inhibitor than APP-IP (Higashi and Miyazaki, 2003a,b, 2008).

The mechanism by which MT1-MMP could promote amyloidogenesis is still elusive. Nevertheless, reminiscent of legumain, MT1-MMP cleavage upstream of the β-cleavage site could facilitate β-secretase cleavage of APP and thus the subsequent production of C99 and Aβ. Not exclusively, MT1-MMP could favor amyloidogenic β-secretase processing by promoting APP sorting into BACE-1-enriched early endosomes (Paumier et al., 2019). Most interestingly, it has been proposed that MT1-MMP could also function as a surrogate β-secretase when BACE-1 is inhibited; this is supported by experiments in HEKswe cells where MT1-MMP is able to restore the basal levels of Aβ, almost abolished in the presence of BACE-1 inhibitor IV (or C3) (Paumier et al., 2019). It should be noted that a β-cleavage site for MT1-MMP has not yet been identified. MT1-MMP replacing BACE-1 to maintain physiological levels of Aβ could be relevant in the event of therapeutic inhibition of BACE-1 if it is assumed that reducing Aβ levels below a physiological threshold could have adverse consequences.

Overall, these data highlight potential new mechanisms in the control of APP processing involving functional interactions of α- and β-secretases with MT1-MMP and MMP-2. It is now time to develop *in vivo* experimental models where genetic (i.e., conditional MT1-MMP knockout mice in transgenic AD mouse) and/or pharmacological (i.e., neutralizing antibodies) manipulation of MT1-MMP activity should further confirm the pathophysiological relevance of the aforementioned *in vitro* data. This includes a functional assessment of the apparent dual activity of MT1-MMP as an amyloidolytic and amyloidogenic enzyme, as well as the *in vivo* validation of APP cleavage and the resulting NTFs and CTFs.

##### MT5-MMP

MT5-MMP was detected in neurons and around senile plaques in the brains of AD patients (Sekine-Aizawa et al., 2001) and it was later suggested to cleave APP, yielding a fragmentation profile similar to that of MT1- and MT3-MMP (Ahmad et al., 2006). More recently, two independent studies highlighted in the same time the involvement of MT5-MMP on APP processing and the subsequent functional consequences (Willem et al., 2015; Baranger et al., 2016b). Willem and collaborators identified by mass spectrometry the VLAN_504_-M_505_ISEPR (APP_695_ numbering) as a cleavage site for MT5-MMP on APP, which was named η-cleavage site (Willem et al., 2015). This site was previously identified for MT1-MMP (Higashi and Miyazaki, 2003b) and MT3-MMP (Ahmad et al., 2006). Intriguingly, the Willem’s study did not identify MT1-MMP as cleaving at this VLAN_504_-M_505_ISEPR/η-site and it was concluded that only MT5-MMP could act as a η-secretase *in vivo* (Willem et al., 2015). η-secretase cleavage of APP generates sAPPη and a residual transmembrane C-terminal APP fragment-η (η-CTF), which accumulates in dystrophic neurites around amyloid plaques in APP/PS1 mice. Using specific antibodies against the neoepitopes of the α-, β- and η-sites, it was observed that BACE-1 inhibition promoted the release of a peptide Aη-α (for amyloid eta-alpha) resulting from the combined action of MT5-MMP and α-secretase. Recombinant Aη-α was able to impair LTP when incubated on primary rat neuronal cultures. In addition, the inhibition of ADAM10 promoted the concerted action of MT5-MMP and BACE-1 to release a shorter peptide, Aη-β, unexpectedly innocuous (Willem et al., 2015). Counter-intuitively, ADAM10 is presented in this work as conveying neurotoxic actions by contributing to the generation of Aη-α together with MT5-MMP. Moreover, the fact that Aη-α levels are exacerbated upon BACE-1 inhibition may raise concerns of possible side effects of therapeutic anti-BACE-1 strategies. Aη-γ peptides, potentially generated by a combined action of MT5-MMP and γ-secretase, were not detected, probably revealing a lack of functional interaction between η- and γ-secretase as it is the case with BACE-1, ADAM10 and γ-secretase (Chen et al., 2015; Liu et al., 2019). Indeed, a recent study has shown that MT5-MMP does not co-purify in the high molecular weight complex formed by BACE-1 and γ-secretase. Replacing the transmembrane domain (TMD) of BACE-1 by that of MT5-MMP did not prevent BACE-1 complex with γ-secretase and Aβ production, suggesting that motifs other than TMD are involved in the formation of the BACE-1/γ-secretase interactions (Liu et al., 2019). Aη-α and Aη-β peptides were both detected in human cerebrospinal fluid (CSF) of healthy and AD patients, but no differences in their levels were observed between groups (Willem et al., 2015). Another study identified a ∼25 kDa η-CTF in CSF, but in this case the peptide content was higher in AD patients with a *PSEN1* familial mutation, in patients with sporadic AD and in Down Syndrome individuals with Alzheimer’s type dementia, compared to age-matched non-demented controls, raising the possibility that η-CTF could be a new disease biomarker (Garcia-Ayllon et al., 2017).

By the same time, our group demonstrated that MT5-MMP induced the production of a soluble APP fragment of sAPP95 in HEKswe, concomitant with elevated levels of C99 and Aβ_40_ (Baranger et al., 2016b). Although the exact nature of sAPP95 produced upon MT1-MMP and MT5-MMP expression needs to be established, it likely corresponds to what was defined as sAPPη by others (Willem et al., 2015). The function of sAPP95/sAPPη is not yet known, but a recent study showed that it binds GABA_*B*_R1a, which also acts as a receptor for sAPPα and sAPPβ, resulting in a reduced probability of presynaptic release (Rice et al., 2019). These findings link MT5-MMP (as well as α- and β-secretases) with the regulation of a major inhibitory neurotransmitter system coupled to Ca^2+^ and K^+^ channels, and expands the range of possible mechanisms by which APP proteolysis may influence changes in synaptic activity occurring at early stages of the disease. Gaining insight into the functional interplay between proteinases and neurotransmission will require precise assessment of the functional specificities of 3 APP NTFs generated by 3 different enzymes sharing a common receptor.

Similarly to MT1-MMP, we found that MT5-MMP could interact with APP and promote its endosomal sorting, thereby providing mechanistic support for MT5-MMP pro-amyloidogenic activity (Baranger et al., 2017a). Moreover, to evaluate the genuine role of MT5-MMP *in vivo*, we generated a bigenic mouse model by crossing 5xFAD mice (Oakley et al., 2006) and MT5-MMP^–/–^ (MT5^–/–^) mice (Komori et al., 2004). Compared to 5xFAD, 5xFAD/MT5^–/–^ mice showed significantly lower levels of sAPP95, validating our *in vitro* observations and APP being an *in vivo* physiological substrate of MT5-MMP (Baranger et al., 2016b). Most important, 5xFAD/MT5^–/–^ mice showed strikingly lower levels of Aβ (soluble Aβ_40_ and Aβ_42_, oligomers and plaques) and C99 at prodromal-like stages of the pathology (Baranger et al., 2016b, 2017a). Moreover, decreased APP processing and amyloidogenesis upon MT5-MMP deficiency were concomitant with fewer reactive microglial cells and astrocytes around amyloid plaques, as well as reduced levels of IL-1β and TNF-α (Baranger et al., 2016b, 2017a). From a functional standpoint, the absence of MT5-MMP prevented deficits in LTP and spatial and working memory observed in 5xFAD mice (Baranger et al., 2016b, 2017a). Most interestingly, some of these beneficial effects were also observed at advanced stages of the pathology in 16-month-old 5xFAD/MT5^–/–^ mice, which presented a better preservation of neuronal networks and synaptic integrity compared to age-matched 5xFAD control mice (Baranger et al., 2016b).

Mechanistically, it is tempting to speculate that MT5-MMP could share with MT1-MMP and legumain proteolytic actions by which cleavage of APP upstream of the β-cleavage site would facilitate the processing of APP by β-secretase, especially in endosomes. Two sets of findings support such hypothesis: (1) bigenic 5xFAD/legumain^–/–^ and 5xFAD/MT5^–/–^ mice both show significant reductions in Aβ burden and glial reactivity, as well as prevention of LTP and learning and memory deficits (Zhang et al., 2015; Baranger et al., 2016b, 2017a), reminiscent of observations made in bigenic 5xFAD/BACE-1^–/–^ or 5xFAD/BACE-1^+/–^ mice (Ohno et al., 2007; Kimura et al., 2010) and (2) MT5-MMP can be internalized in endosomes (Wang et al., 2004; Baranger et al., 2017a) and thus promote endosomal APP sorting (Baranger et al., 2017a). In such hypothetical model, the first cleavage by MT5-MMP at the η-site could have place on the membrane and be followed by joint internalization with the APP transmembrane breakdown product (CTF-30/η-CTF). It is noteworthy that MT5-MMP trafficking involves interactions between the last three residues at carboxyl terminus and Mint3, a protein that contains two type III PDZ domains (Wang et al., 2004). Remarkably, Mint3 can also function as an adaptor protein to promote APP export from the Golgi complex (Caster and Kahn, 2013). These observations highlight Mint3 as a potential molecular bridge between MT5-MMP and APP, which could facilitate their interactions in the context of traffic-based mechanisms for the control C99/Aβ production. This is consistent with the increasing importance granted to traffic regulation in the amyloidogenic process, but also as means of exchanging relevant biofactors between cells. In this vein, η-CTF was detected in extracellular vesicles released by N2a neuroblastoma cells expressing APP Swedish. The vesicles were uptaken by neurons, suggesting the possibility that η-CTF could function as a vector for intercellular pathogenic propagation (Laulagnier et al., 2018).

## Secretase Inhibitors/Modulators Can Be Linked to AD

An indirect way to assess the physiological impact of the new APP processing enzymes is through the study of their endogenous inhibitors, assuming that the control of proteolytic activities is an important component of the final proteolytic balance. Such an evaluation is not an easy task because the inhibitory selectivity is not always completely elucidated, as is often the case with metalloproteinases, which have a highly conserved catalytic site. In general, the available literature on proteinase inhibitors and AD remains essentially correlative and the link between both is to date inconclusive or, at best, speculative. In this context, the levels of fetuin-A, an inhibitor of meprin-β, were found to be reduced in CSF of AD patients compared to healthy individuals (Puchades et al., 2003). In addition, a polymorphism in the promotor sequence of the fetuin-A encoding gene has been associated with late onset AD (LOAD) (Geroldi et al., 2005), and lower plasma levels of fetuin-A correlated with severe to mild cognitive impairment (Smith et al., 2011). Likewise, a polymorphism in the cystatin C gene (CST3) was significantly associated with LOAD patients, while lower levels of this legumain inhibitor were found in their plasma (Chuo et al., 2007). Other studies have shown that overexpression of cystatin C in brains of APP-transgenic mice reduces amyloid plaque formation (Kaeser et al., 2007). A potential protective role of cystatin C in AD could be related to its ability to bind soluble Aβ and thus prevent Aβ deposition (Mi et al., 2007). A clear link between high cholesterol levels and the pathogenesis of AD has not yet been confirmed, but patients taking statins, a cholesterol-lowering drug, are still less likely to develop the disease (Eckert et al., 2005; Fonseca et al., 2010; Wood et al., 2014). In this context, it has been shown that high cholesterol levels inhibit RHBDL4 activity, which has been correlated with an increase in the formation of Aβ (Paschkowsky et al., 2018). For TIMP-2, the main inhibitor of MT-MMPs, clinical data are rather inconsistent. For example, one study showed no change in plasma TIMP-2 concentrations in patients with AD compared to healthy individuals (Lorenzl et al., 2003b), while in the CSF, two independent studies showed no difference (Mroczko et al., 2014) or increased (Lorenzl et al., 2003a) TIMP-2 levels in AD patients. More recently, Duits and collaborators reported decreases in TIMP-2 (and also TIMP-1) in the CSF of AD patients with microbleeds (Duits et al., 2015). Pre-clinical work has reported TIMP-2 as a possible anti-aging factor as it promotes neuronal plasticity and hippocampal-dependent cognition in aging mice (Castellano et al., 2017). In this study, the authors used parabiosis experiments to demonstrate that plasma from young donor mice (3 months) enriched with TIMP-2 reversed cognitive deficits in elderly mice (18 months). The confirmation that TIMP-2 was responsible for the observed effects was obtained in experiments where the plasma of young TIMP-2 knockout mice failed to rescue the aged phenotype. It is noteworthy that the levels of many immune and trophic factors were upregulated in plasma from TIMP-2 knockout mice, indicating a possible regulatory role of TIMP-2 on molecules that influence the functional cross talk between the CNS and peripheral tissues. It is therefore tempting to speculate on TIMP-2 as a pleiotropic “rejuvenating” factor with anti-MMP activities and, as shown earlier, with anti-mitotic and neural cell-differentiation promoting effects independent of MMP-inhibitory activity (Perez-Martinez and Jaworski, 2005). The idea of a “global” anti-aging factor is particularly appealing because aging is by far the main risk factor for AD and other degenerative disorders of nervous and non-nervous tissues.

Overall, the available partial data on proteinase inhibitors highlight the need for more functional studies. These should confirm their potential in modulating proteolytic balance or non-proteinase-dependent actions as a means of deciphering new AD pathophysiological pathways and developing new therapeutic strategies.

## Concluding Remarks and Future Directions

The importance of APP proteolysis in AD mechanisms is not in doubt. The emergence of new APP cleaving enzymes significantly broadens the scope of study and makes evident the need for a comprehensive understanding of the complex molecular processes that encompasses APP proteolysis and the subsequent range of beneficial/pathological effects. Gaining insight in such processes will depend on our ability to determine the nature and functions of new APP fragments generated by these enzymes in a given cell type, subcellular compartment or stage of the disease, in particular at early stages where they could affect neurotransmitter release, synaptic function or incipient neuroinflammation. APP fragments can also be a new source of inspiration for developing therapeutic approaches based on blocking or promoting their formation. This may intrinsically require specific modulation of a given proteolytic cleavage, which turns out to be extremely difficult when the targeted proteinase has many substrates. This is the case for BACE-1 or γ-secretase, with dozens of substrates identified to date (Haapasalo and Kovacs, 2011; Kuhn et al., 2012; Zhou et al., 2012; Vassar et al., 2014). The list of substrates for most of the emerging proteinases discussed here is still relatively short and could therefore offer a therapeutic advantage in this respect. Time will tell whether the number of substrates will increase as new studies are conducted. Alternatively, APP proteolytic fragments may become full-fledged targets and their functions inhibited or potentiated, thus making it unnecessary to specifically inhibit the proteolytic processes that produce them. This option logically relies on a thorough knowledge of the biological functions of these fragments but also of the structural APP features. An additional alternative to modulating proteinase catalytic activity is to gain insight into the trafficking of APP and its metabolites. Mounting evidence indicates that the way these molecules circulate in the cell (or between cells) affects their functions and their availability for proteolytic processing. Similarly, understanding the cellular routing of proteinases may also be crucial to better target them. Beyond proteolytic activity, proteinases can also exert non-proteolytic actions through domains of interaction with other proteins; the question arises of the importance that these phenomena could have in the pathogenesis of AD and the new avenues they open to targeting domains other than the catalytic domain. This idea works as a mirror when it comes to the implications of proteinase inhibitors (e.g., TIMPs), which are known to control cell cycle, cell migration or cell fate also independent of anti-proteinase activities (Baranger et al., 2014). Since inhibitors are usually circulating proteins, they could favor communication between the CNS and peripheral systems (e.g., cardiovascular and immune systems), a process on which brain function and aging may depend.

In summary, this review highlights APP as a central molecule in the pathophysiology of AD and as a common substrate for MT-MMPs and other emerging proteinases. However, in the context of a more holistic approach to the disease that goes beyond APP/Aβ, we anticipate that in the coming years proteolytic and non-proteolytic interaction models will be established between proteinases, inhibitors and their partner proteins to continue to reveal the diversity of pathogenic pathways in AD and to learn how better control them.

## Author Contributions

All the authors discussed, wrote and approved the manuscript. KB and SR supervised the writing of the manuscript.

## Conflict of Interest Statement

The authors declare that the research was conducted in the absence of any commercial or financial relationships that could be construed as a potential conflict of interest.

## Abbreviations

5xFAD: transgenic mouse model of AD bearing 3 familial mutations on human *APP* and 2 on *PSEN1* genes
AD: Alzheimer’s disease
ADAM: a disintegrin and metalloproteinase
AICD: APP intracellular domain
AMPA: α-amino -3-hydroxy-5-methyl-4- isoxazolepropionic acid
APLP1/2: amyloid precursor like protein 1/2
APOE: apolipoprotein E
APP: amyloid-beta precursor protein
APP-IP: APP-derived inhibitor peptide
A β: amyloid-beta peptide
BACE-1: beta-site APP cleaving enzyme 1
C99/C83: APP-CTF of 99/83 amino acids
CSF: cerebrospinal fluid
CST3: cystatin C encoding gene
CXCL12: C-X -C motif chemokine ligand 12
DR6: death receptor 6
ECM: extracellular matrix
GABA: gamma-aminobutyric acid
GluA1/A2: glutamate A1/A2
GPI: glycosylphosphatidyl inositol
HEKswe: Human Embryonic Kidney cells 293 stably expressing *APP* with the familial Swedish mutation
IL-1 β: interleukin-1 beta
IL-8: interleukin-8
iPS: induced pluripotent stem cells
LOAD: late onset Alzheimer’s disease
LTP: long-term potentiation
mEPSCs: mini excitatory postsynaptic currents
mIPSCs: mini inhibitory postsynaptic currents
MMP: matrix metalloproteinase
MT-MMP: membrane-type matrix metalloproteinase
N/CTF: N-terminal or C-terminal APP fragments generated by APP-cleaving enzymes
NFT: neurofibrillary tangles
NMDA: *N*-methyl -D-aspartate
PARL: presenilin associated rhomboid like
PP2A: protein phosphatase 2
PS1/2: presenilin 1 and 2
PSD95: postsynaptic density protein 95
RHBDL4: rhomboid-like protein-4
sAPP α / β: soluble APP α / β
SDF1 α: stromal cell-derived factor 1
SLPI: secretory leukocyte proteinase inhibitor
TIMP: tissue inhibitor of MMPs
TMD: transmembrane domain
TNF- α: tumor necrosis factor alpha
VEGF: vascular endothelial growth factor.
